# The transcription factor OsbHLH035 mediates seed germination and enables seedling recovery from salt stress through ABA-dependent and ABA-independent pathways, respectively

**DOI:** 10.1186/s12284-018-0244-z

**Published:** 2018-09-10

**Authors:** Hung-Chi Chen, Wan-Hsing Cheng, Chwan-Yang Hong, Yu-Sen Chang, Men-Chi Chang

**Affiliations:** 1 Department of Agronomy, National Taiwan University, No. 1, Section 4, Roosevelt Road, Taipei, Taiwan, Republic of China; 2 Institute of Plant and Microbial Biology, Academia Sinica, Taipei, Taiwan, Republic of China; 3 Department of Agricultural Chemistry, National Taiwan University, Taipei, Taiwan, Republic of China; 4 Department of Horticulture and Landscape Architecture, National Taiwan University, Taipei, Taiwan, Republic of China

**Keywords:** ABA, bHLH, OsHKT, Salt stress, Transcription factor

## Abstract

**Background:**

Many transcription factors (TFs), such as those in the basic helix-loop-helix (bHLH) family, are important for regulating plant growth and plant responses to abiotic stress. The expression of *OsbHLH035* is induced by drought and salinity. However, its functional role in rice growth, development, and the salt response is still unknown.

**Results:**

The bHLH TF *OsbHLH035* is a salt-induced gene that is primarily expressed in germinating seeds and seedlings. Stable expression of GFP-fused OsbHLH035 in rice transgenic plants revealed that this protein is predominantly localized to the nucleus. *Osbhlh035* mutants show delayed seed germination, particularly under salt-stress conditions. In parallel, abscisic acid (ABA) contents are over-accumulated, and the expression of the ABA biosynthetic genes *OsABA2* and *OsAAO3* is upregulated; furthermore, compared with that in wild-type (WT) seedlings, the salt-induced expression of *OsABA8ox1*, an ABA catabolic gene, in germinating *Osbhlh035* mutant seeds is downregulated. Moreover, *Osbhlh035* mutant seedlings are unable to recover from salt-stress treatment. Consistently, sodium is over-accumulated in aerial tissues but slightly reduced in terrestrial tissues from *Osbhlh035* seedlings after salt treatment. Additionally, the expression of the sodium transporters *OsHKT1;3* and *1;5* is reduced in *Osbhlh035* aerial and terrestrial tissues, respectively. Furthermore, genetic complementation can restore both the delayed seed germination and the impaired recovery of salt-treated *Osbhlh035* seedlings to normal growth.

**Conclusion:**

OsbHLH035 mediates seed germination and seedling recovery after salt stress relief through the ABA-dependent and ABA-independent activation of *OsHKT* pathways, respectively.

**Electronic supplementary material:**

The online version of this article (10.1186/s12284-018-0244-z) contains supplementary material, which is available to authorized users.

## Background

Salt stress severely affects plant growth and development. In fact, more than 20% of the land used for agriculture is of poor quality due to soil salinity (FAO, http://www.fao.org/home/en/). The adverse effects of salt stress on plant growth and development can be attributed to initial osmotic dehydration and subsequent ion toxicity. Osmotic stress signaling is known to be transduced via both ABA-dependent (e.g., mitogen-activated protein kinases [MAPKs]) and ABA-independent (e.g., via the OsDREB1 and OsDREB2 TFs) pathways (Kumar et al. 2013). Additionally, the maintenance of cellular ion homeostasis is important for plant survival under salt stress, especially in response to high cytosolic K^+^/Na^+^ ratios. In fact, many transporters, including HKTs (high-affinity potassium transporters), NHXs (Na^+^/H^+^ exchangers), and SOS (salt overly sensitive), act collectively to regulate intracellular sodium levels (Deinlein et al. 2014). The regulation of *HKTs* expression is critical for mediating salt stress tolerance in crops (Hamamoto et al. 2015). HKT transporters can be divided into two subfamilies. Subfamily I *HKT* genes (*HKT1s*) encode Na^+^-selective transporters, which are mostly located at the plasma membrane in xylem parenchyma cells. The functional role of HKT1s is responsible for retrieving Na^+^ from the xylem sap, which prevent aerial tissues from Na^+^ over-accumulation and toxicity. Subfamily II HKT transporter (HKT2) is involved in uptake of Na^+^ from the external medium (soil) when K^+^ is limiting. Therefore, when plants are exposed to salt, the phytohormone ABA, TFs, and transporters act as key components that integrate cellular signal transduction and stress-responsive gene expression to confer salt tolerance.

ABA plays a critical role in regulating seed dormancy, germination, and stress tolerance (Karssen et al. 1983; Koornneef et al. 1989; Groot and Karssen 1992; Qin and Zeevaart 2002; Finkelstein et al. 2008). To date, most genes related to ABA metabolism have been cloned and characterized (for review, see Taylor et al. 2000; Seo and Koshiba 2002; Nambara and Marion-Poll 2005; Ye et al. 2012). The first committed step of ABA biosynthesis involves the oxidative cleavage of a C_40_ 9-*cis*-epoxycarotenoid, such as 9’-*cis*-neoxanthin or 9-*cis*-violaxanthin, to a C_15_ xanthoxin (the primary precursor) and a C_25_ by-product, which is catalyzed in plastids by 9-*cis*-epoxycarotenoid dioxygenase (NCED) (Schwartz et al. 2003; Xiong and Zhu 2003). At least 5 *NCED* genes have been documented in *Arabidopsis*, and 3 have been reported in rice (Tan et al. 2003; Welsch et al. 2008). Of the five *AtNCEDs* (*AtNCED2*, *3*, *5*, *6*, and *9*), only *AtNCED3* is significantly induced by drought stress, and it controls the levels of endogenous ABA under drought-stress conditions (Iuchi et al. 2001). In rice, the expression of *OsNCED3, OsNCED4,* and *OsNCED5* was induced after 1 h of salt stress and closely correlated with the endogenous ABA contents in roots (Welsch et al. 2008). ABA biosynthesis subsequently involves the conversion of xanthoxin to abscisic aldehyde (an intermediate), and the final steps of ABA production are catalyzed in the cytosol by AtABA2 and AtAAO3 in *Arabidopsis* (Seo et al. 2000; Rook et al. 2001; Cheng et al. 2002; Gonzalez-Guzman et al. 2002). For full catalytic activity, AtAAO3 requires a molybdenum cofactor, which is derived from AtABA3 function (Bittner et al. 2001; Xiong et al. 2001). Indeed, loss-of-function mutations in *AtABA2*, *AtAAO3*, and *AtABA3* lead to ABA deficiency and early germination. Cellular ABA homeostasis is regulated by both the biosynthesis and degradation of ABA. ABA catabolism occurs via two main types of chemical reactions, hydroxylation and conjugation, with the hydroxylation of ABA at position C-8’ being the key regulatory step (Kushiro et al. 2004; Saito et al. 2004; Nambara and Marion-Poll 2005). *Arabidopsis* and rice possess 4 and 3 known *ABA8ox* genes, respectively (Kushiro et al. 2004; Saito et al. 2004; Saika et al. 2007). The four *AtABA8ox* genes, also known as *CYP707A*s (*CYP707A1*, *2*, *3*, and *4*), display complex spatiotemporal expression patterns and are important for many physiological processes. For example, *CYP707A1* and *CYP707A2* but not *CYP707A3* or *CYP707A4* are responsible for relieving the ABA-mediated inhibition of seed germination (Okamoto et al. 2006). These *CYP707A*s are induced by osmotic stress presumably because the maintenance of endogenous ABA homeostasis under stress is necessary for plant survival (Saito et al. 2004). Although the biochemical and physiological roles of these ABA metabolic genes have been well characterized and documented, the mechanisms of their transcriptional regulation are still poorly understood.

Deciphering the roles of TFs is essential for revealing abiotic stress tolerance mechanisms in plants (Singh et al. 2002). In fact, several different types of TFs have been demonstrated to be involved in regulating abiotic stress responses (for review, see Nakashima et al. 2009; Todaka et al. 2012). For example, AtbHLH116/ICE1-*CBF3/DREB1A* [*AtERF#031* (At4g25480)] acts in the cold-responsive signaling pathway via an ABA-independent pathway (Chinnusamy et al. 2003); ABI3, 4, and 5 (B3, AP2/ERF, and bZIP, respectively) act in the osmotic stress response pathway via an ABA-dependent pathway (Giraudat et al. 1992; Finkelstein et al. 1998; Finkelstein and Lynch 2000); and SUB1A (AP2/ERF) acts in submergence- and drought-responsive pathways (Fukao et al. 2011). Additionally, many TF types, including bHLH (Zhou et al. 2009; Li et al. 2010), WRKY (Tao et al. 2011), NAC (Hu et al. 2006), and AP2/ERF (Schmidt et al. 2013), are involved in the regulation of salt stress tolerance in rice. Approximately 7% of plant genes encode TFs. The *Arabidopsis* and rice (*Oryza sativa subsp. japonica*) genomes contain 2016 and 2424 TFs, respectively, that can be categorized into 58 and 56 families based on their DNA binding domains (Zhang et al. 2011). Basic helix-loop-helix (bHLH) TFs comprise the second largest TF family in plants and can affect many developmental and physiological processes (Feller et al. 2011). To date, at least 167 *Arabidopsis* and 177 rice *bHLH*s have been identified (Li et al. 2006; Carretero-Paulet et al. 2010). These genes are classified based on the proteins they encode, which usually contain a conserved bHLH domain (approximately 60 amino acids long), with the exception of a few atypical *bHLH* genes known as *HLH*s that lack the basic region (Li et al. 2006). A typical bHLH domain comprises two functionally distinct regions: a basic region for DNA binding and an HLH region for protein homodimerization or heterodimerization (Massari and Murre 2000; Toledo-Ortiz et al. 2003; Feller et al. 2011). Thus, HLH proteins can heterodimerize with bHLH proteins, thereby disrupting bHLH-bHLH interactions and preventing DNA binding (Sun et al. 1991; Toledo-Ortiz et al. 2003). bHLH proteins can recognize two types of target *cis*-acting elements: the E-box (5’-CANNTG-3’) and the G-box (5’-CACGTG-3’). Previous studies have demonstrated that several AtbHLHs are involved in regulating abiotic stress responses. AtbHLH116/ICE1 regulates the expression of *CBF3/DREB1A*, an AP2/ERF TF, that provides freezing tolerance through an ABA-independent pathway that is also known as the ICE-CBF regulon (Chinnusamy et al. 2003). OrbHLH1 and OrbHLH2, two ICE-like proteins in wild rice (*Oryza rufipogon*), positively regulate salt-stress responses in transgenic *Arabidopsis* through an ICE/CBF-independent and ABA-independent pathway, respectively (Zhou et al. 2009; Li et al. 2010). More recently, AtbHLH122 has been shown to confer tolerance to drought, salt, and osmotic stresses through an ABA-dependent pathway via the transcriptional repression of *CYP707A3*, thereby blocking ABA degradation (Liu et al. 2014). Notably, only approximately 10% of *OsbHLHs* have been characterized, in contrast to 38% of *AtbHLH*s (Heang and Sassa 2012).

To investigate which uncharacterized *OsbHLH*s may also be involved in the regulation of abiotic stress responses, we analyzed the gene expression profiles from publicly available microarray data (GSE6901) in the NCBI-GEO database, and several abiotic stress-responsive *OsbHLHs* were found, including *OsbHLH035* (Additional file 1: Figure S1). In this study, we examined the roles of *OsbHLH035* in rice during the germination and seedling stages. Using a reverse genetic approach with a *Tos17*-tagged mutant line, we found that *OsbHLH035* mediates germination and confers recovery after salt stress relief through ABA-dependent and ABA-independent pathways, respectively.

## Results

### Characterization of the OsbHLH035 gene and its Tos17-tagged mutant line, NG7221

**Fig. 1:**
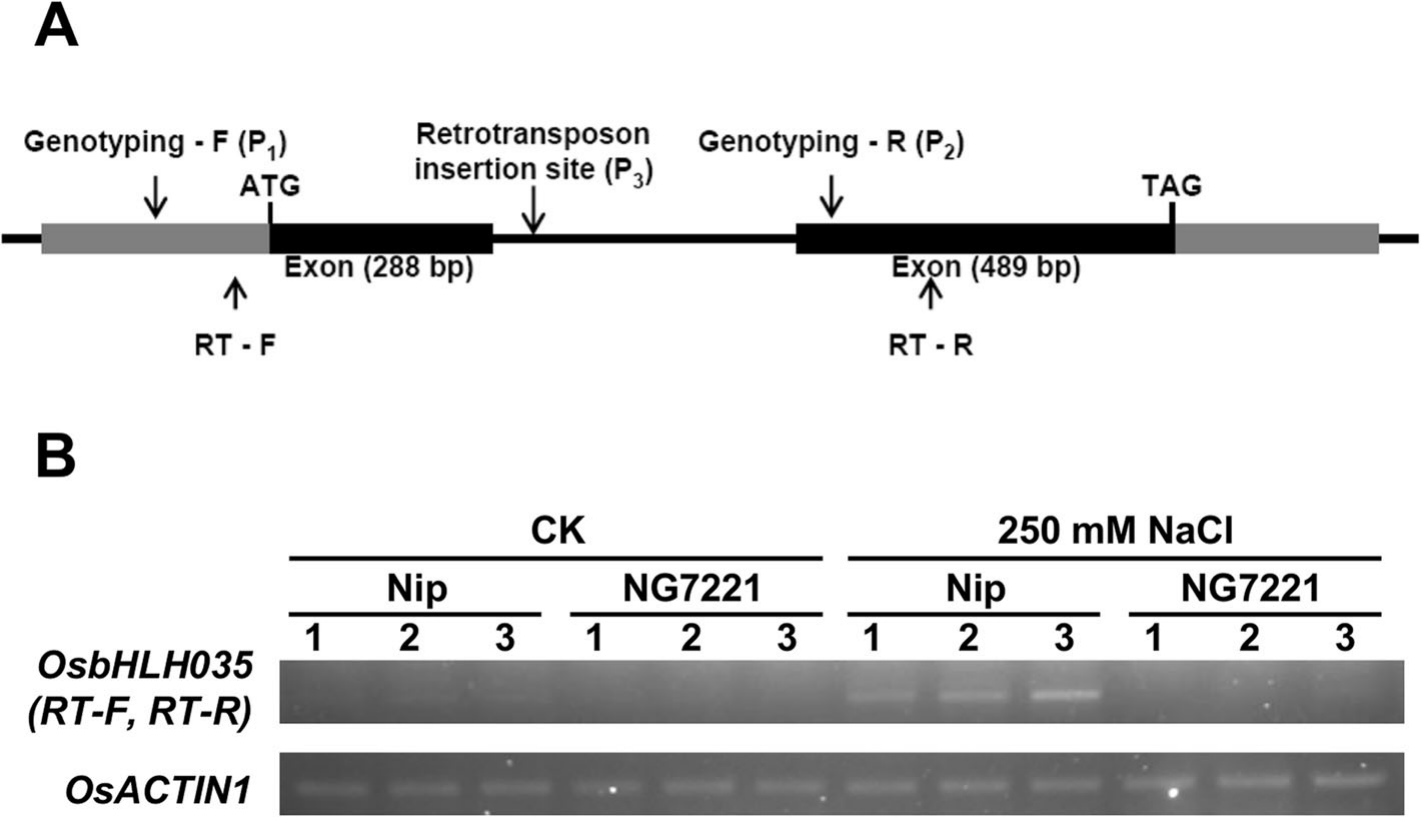
*OsbHLH035* expression is induced by salt and is abolished in the NG7221 line. **a** The structure of the *OsbHLH035* gene and the retrotransposon insertion site. **b** *OsbHLH035* expression patterns in aerial tissues from third-leaf-stage WT and NG7221 seedlings. The seedlings were grown on basal medium for 13 days and then transferred to basal medium containing 0 (CK) or 250 mM NaCl for an additional day. The primer positions and sequences are shown in (**a**) and Additional file 2: Table S1. Arabic numerals in (**b**) represent three independent biological replicates within each genotype

In silico analyses of gene expression profiles revealed that *OsbHLH035* gene expression is upregulated by drought and salt treatments (Additional file 1: Figure S1). The *OsbHLH035* gene contains two exons, and the encoded protein, which putatively binds G-boxes, contains a typical bHLH domain (residues 64 to 113) (Fig. 1a, Additional file 1: Figure S2A). To verify the microarray data, we compared the expression pattern of *OsbHLH035* in aerial tissues from WT rice (*Oryza sativa* L. cv. Nipponbare, Nip) under normal and salt-treated conditions using RT-PCR. As shown in Fig. 1b, *OsbHLH035* transcripts were barely detectable in aerial tissues under normal conditions, whereas they were present under salt-treated conditions. These data indicate that *OsbHLH035* is reliably induced by salt stress in rice seedlings.

To investigate the roles of *OsbHLH035* in plant growth and salt-stress responses, we searched the rice *Tos17* insertion mutant database and identified a retrotransposon insertional *Osbhlh035* mutant line, NG7221. Based on its annotation, the retrotransposon is located in an *OsbHLH035* gene intron (Fig. 1a, Additional file 1: Figure S2B). Homozygous mutants were identified from the segregating population by genomic DNA genotyping (Additional file 1: Figure S2C). RT-PCR analysis showed that *OsbHLH035* transcripts were absent in aerial tissues from third-leaf-stage NG7221 seedlings under both normal and salt-treated conditions (Fig. 1b). Therefore, the NG7221 line is used as an *Osbhlh035* mutant in this study.

### Spatiotemporal OsbHLH035 expression and OsbHLH035 localization

**Fig. 2:**
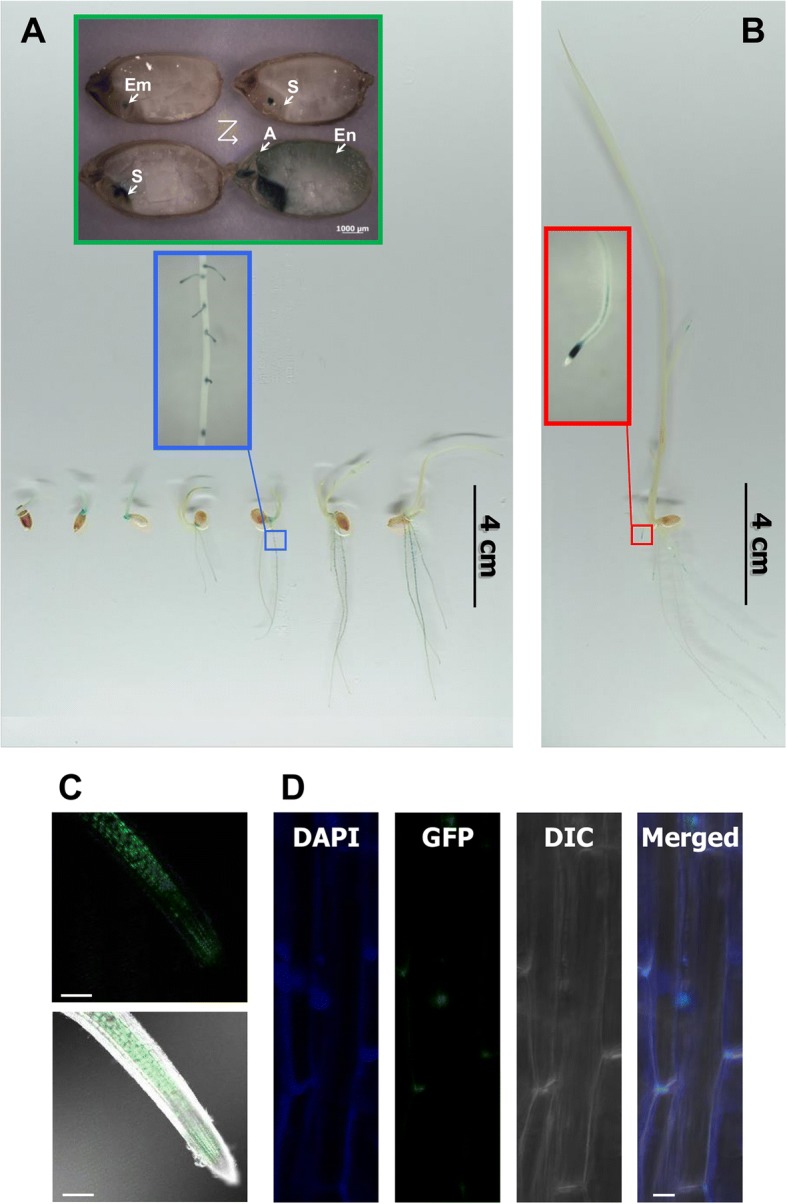
The spatiotemporal expression of *OsbHLH035* and the subcellular localization of GFP-OsbHLH035 in stable rice transformants. **a** The germinating seeds and post-germination-stage seedlings. The Z-scheme represents the staining order of the water-imbibed transgenic seeds, which were collected within 2 days. Em, Embryo; S, Scutellum; A, Aleurone layer; and En, Endosperm. **b** A third-leaf-stage seedling. **c** The presence of GFP-OsbHLH035 in the root tip. The upper panel is fluorescence image; the lower panel is overlaid with transmitted light image. Scale bars, 200 μm. **d** GFP-fused OsbHLH035 protein is localized with the DAPI, a nuclear affinity dye, in rice cells. Scale bars, 5 μm

Analyzing spatiotemporal gene expression patterns and protein localization can provide valuable insights into gene function. Thus, we monitored *OsbHLH035* gene expression by detecting β-glucuronidase (GUS) signals in *OsbHLH035::GUS* transgenic plants from the germination stage until the third-leaf stage. In the germinating seeds, time-dependent expression of *GUS* was observed in the embryo, scutellum, aleurone layer, and endosperm after imbibition (Fig. 2a). In the aerial tissues, *GUS* was expressed in the hypocotyl and coleoptile at the postgermination stage but was difficult to detect in the third-leaf-stage seedlings (Fig. 2a, b). In terrestrial tissues, GUS signals were present in the root tip, vascular tissue, and lateral root initiation sites of both postgermination and third-leaf-stage seedlings (Fig. 2a, b).

To validate the spatial expression of *OsbHLH035* and the subcellular localization of OsbHLH035 protein in rice, an *OsbHLH035::GFP-OsbHLH035* construct was transformed into the *Osbhlh035* mutant via stable *Agrobacterium*-mediated transformation. Similar to its transcriptional expression pattern, the GFP-OsbHLH035 fusion protein was present in the root tip and vascular tissue (Fig. 2b vs. c) and was predominantly localized to the nucleus in rice cells and calli (Fig. 2d, Additional file 1: Figure S3).

### Excess ABA leads to reduced germination rates in Osbhlh035 mutants

**Fig. 3:**
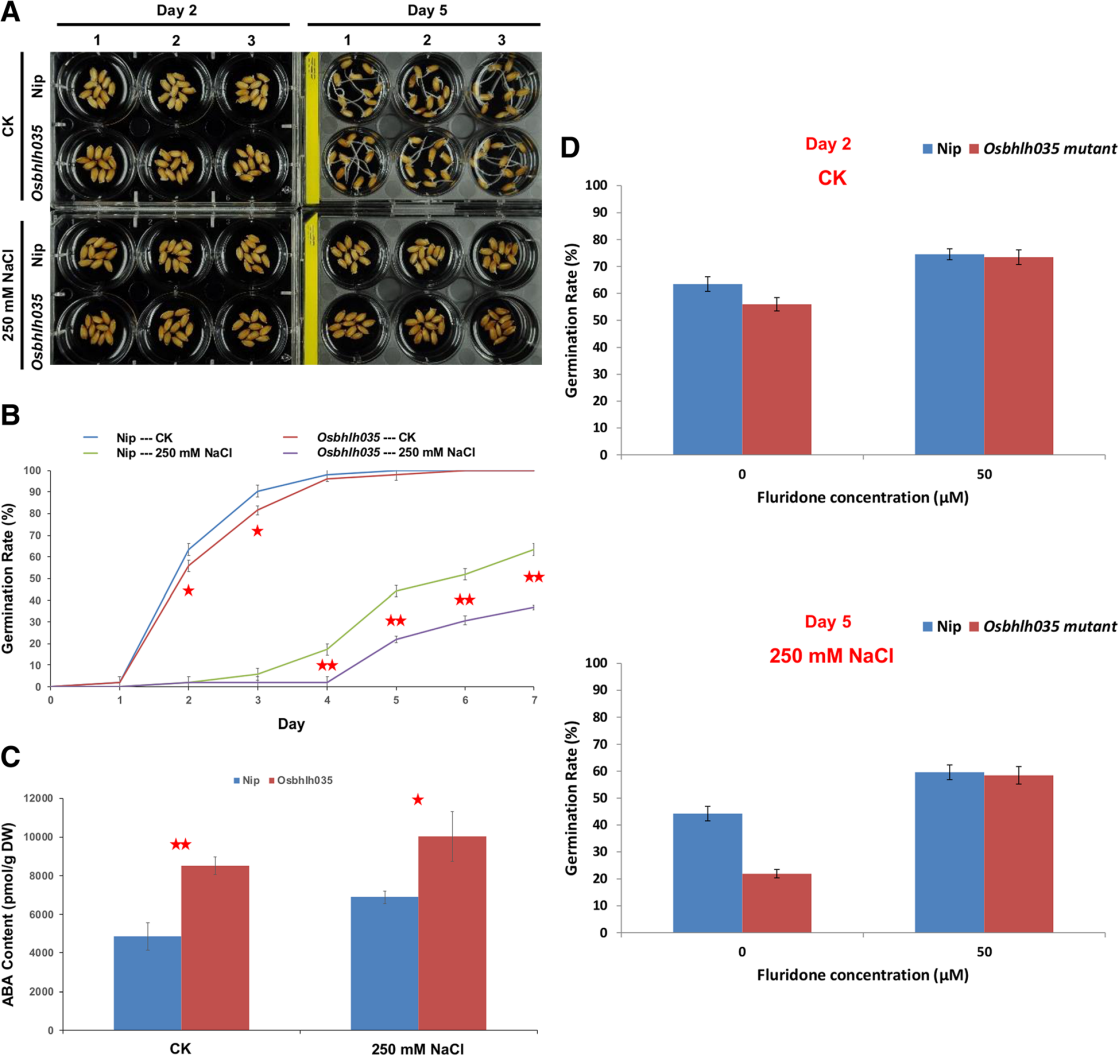
The delayed-germination phenotype of *Osbhlh035* mutants is accompanied by the excess accumulation of endogenous ABA. **a** The germination of WT and *Osbhlh035* seeds under normal and salt-treated conditions at 2 and 5 days after imbibition. Arabic numerals represent three independent biological replicates, and the seeds in each independent biological replicate were harvested from an individual rice plant. **b** The germination rates of WT and *Osbhlh035* seeds under normal and salt-treated conditions. All germination rates represent the mean ± SD of two independent experiments performed with three independent biological replicates; 100 seeds were used in each independent biological replicate. **c** The endogenous ABA contents in both germinating WT and *Osbhlh035* seeds grown under normal and salt-treated conditions on day 5. **d** The delayed-germination phenotype of *Osbhlh035* mutants can be rescued by fluridone. The germination rates of WT and *Osbhlh035* seeds under normal conditions at day 2 (upper panel) and salt-treated conditions at day 5 (lower panel). The seeds were grown on basal medium containing 0 (normal conditions, CK) or 250 mM NaCl (salt-treated conditions) supplemented with or without 50 μM fluridone. Samples for the ABA ELISA were harvested on day 5. Asterisks indicate significant differences in comparison with WT (*P < 0.05 and **P < 0.01) based on Student’s *t*-test

To investigate whether OsbHLH035 plays a role in regulating germination, we compared the germination rates of both WT and *Osbhlh035* plants under normal and salt-treated conditions. Under normal conditions, the WT germination rates were 63.46% and 90.38% on days 2 and 3, respectively; however, the *Osbhlh035* germination rates were reduced to 55.93% and 81.64%, respectively (Fig. 3a, b). Nevertheless, almost all of the WT and the *Osbhlh035* seeds had germinated by day 4. Under salt-treated conditions, the WT germination rates were 17.31%, 44.23%, 51.92%, and 63.46% on days 4, 5, 6, and 7, respectively; however, they were 1.92%, 21.96%, 30.58%, and 36.75%, respectively, in the *Osbhlh035* mutant. Notably, when normalized to the corresponding WT germination rates, the *Osbhlh035* germination rates under normal conditions were reduced by 11.87% and 9.67% on days 2 and 3, respectively. However, the *Osbhlh035* germination rates in the salt-treated conditions were reduced by 88.91%, 50.35%, 41.1%, and 42.09% on days 4, 5, 6, and 7, respectively. Taken together, these data indicate that OsbHLH035 may promote rice seed germination, especially under salt stress.

Because seed germination is inhibited by ABA, which antagonizes gibberellin signaling, we then measured the endogenous ABA contents in germinating WT and *Osbhlh035* seeds. After 2 days under normal conditions, the ABA levels in germinating *Osbhlh035* seeds were approximately 1.84-fold higher than those in WT seeds, and the corresponding germination rate in the *Osbhlh035* mutant was lower than in WT (Fig. 3b, Additional file 1: Figure S4). Additionally, salt-induced ABA accumulation was observed in germinating WT and *Osbhlh035* seeds after 5 days under salt-treated conditions (Fig. 3c). However, the ABA levels were approximately 1.45-fold higher in germinating *Osbhlh035* seeds than in WT seeds, and the corresponding germination rate of the *Osbhlh035* mutant was also lower than that of WT (Fig. 3b, c). To validate that the delayed-germination phenotype of *Osbhlh035* mutants is due to excess ABA accumulation, the biosynthetic inhibitor fluridone was used in further tests. Indeed, the germination rates of WT and *Osbhlh035* seeds were not statistically significantly different after 2 days under normal conditions or 5 days under salt-treated conditions in the presence of fluridone (Fig. 3d). Taken together, these data suggest that OsbHLH035 is involved in the repression of ABA-inhibited germination.

### Expression of ABA metabolic genes in germinating WT and Osbhlh035 seeds

**Fig. 4:**
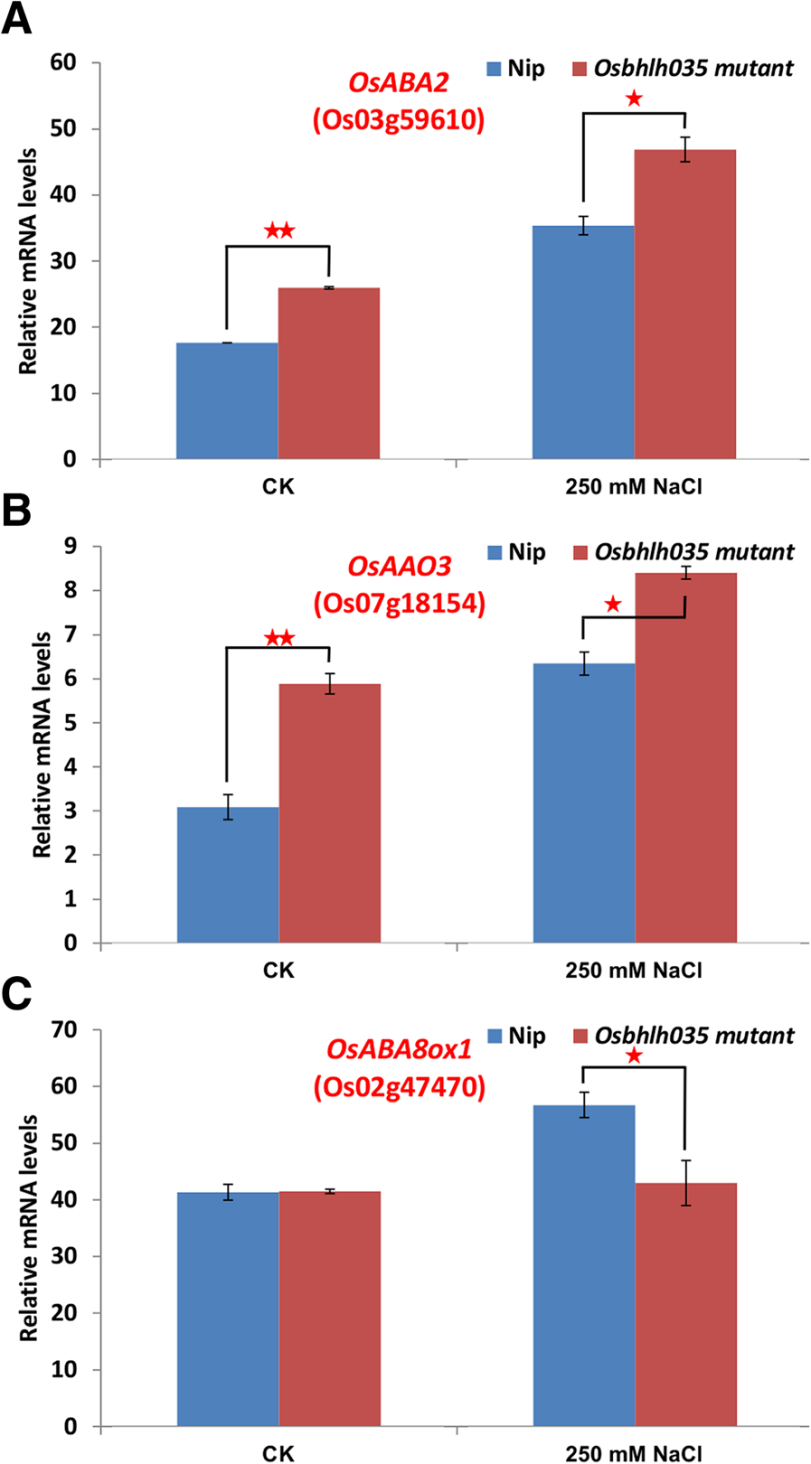
The expression of ABA metabolic genes in both germinating WT and *Osbhlh035* seeds. ABA over-accumulation in germinating *Osbhlh035* seeds is due to the upregulation of the biosynthetic genes *OsABA2* (**a**) and *OsAAO3* (**b**) and abolition of salt-induced *OsABAox1* (**c**) expression. The experimental materials and methods are the same as those described in Fig. 3. Samples for the q-PCR assay were harvested on day 5. Asterisks indicate significant differences in comparison with WT (*P < 0.05 and **P < 0.01) based on Student’s *t*-test

Because ABA metabolic genes have been well characterized in rice, we used q-PCR to investigate the expression profiles of these genes in germinating WT and *Osbhlh035* seeds. In germinating WT seeds, the expression levels of the ABA biosynthetic genes *OsABA2* and *OsAAO3* under salt-treated conditions were approximately 2.01- and 2.06-fold, respectively, higher than those under normal conditions (Fig. 4a, b). However, the *OsABA2* and *OsAAO3* expression levels in germinating *Osbhlh035* seeds were also higher than those in germinating WT seeds under either normal or salt-treated conditions. Moreover, the expression levels of the ABA catabolic gene *OsABA8ox1* in germinating WT and *Osbhlh035* seeds were comparable under normal conditions; however, the salt-induced expression of *OsABA8ox1* was abolished in germinating *Osbhlh035* seeds (Fig. 4c). These results agreed with the corresponding ABA levels detected under these conditions (Fig. 3c vs. Fig. 4). Taken together, these data reveal that the accumulation of excess ABA in germinating *Osbhlh035* seeds may be caused not only by elevated ABA biosynthesis but also by reduced ABA catabolism under salt-treated conditions.

### OsbHLH035 is not involved in regulating plant growth during the seedling stage

**Fig. 5:**
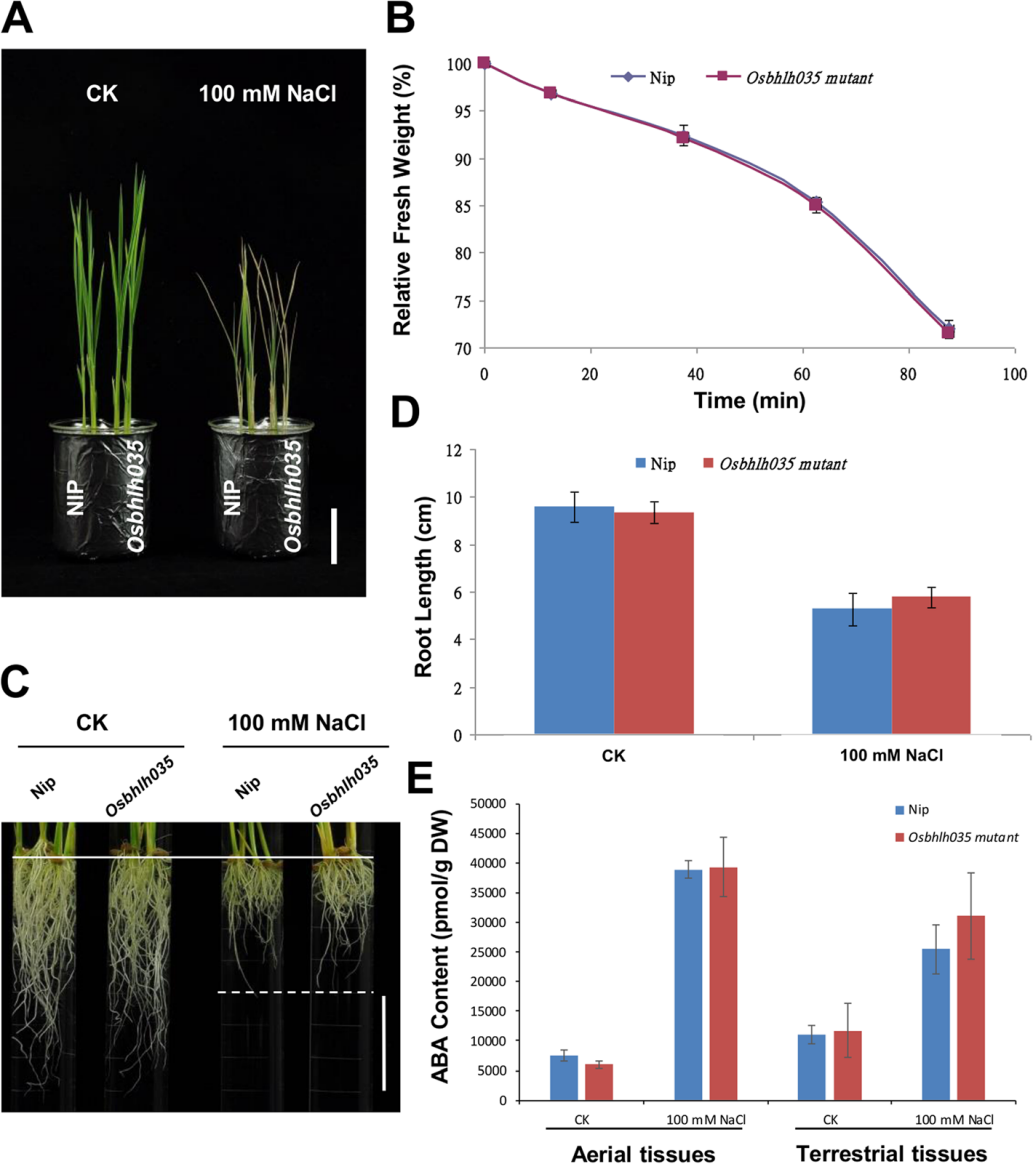
A comparison of the growth patterns, salt responses, and ABA contents in both WT and *Osbhlh035* seedlings under normal (CK) and salt-treated (100 mM NaCl) conditions. The seedlings were grown on basal medium for 7 days and then transferred to basal medium supplemented with 0 (CK) or 100 mM NaCl for an additional 7 days. **a** The growth patterns and salt responses of 14-day-old WT and *Osbhlh035* aerial tissues. **b** The rates of water loss in 14-day-old WT and *Osbhlh035* aerial tissues. **c-d** Characterization and quantification of root growth in both WT and *Osbhlh035* seedlings. **e** The ABA contents in 14-day-old WT and *Osbhlh035* seedlings. Scale bars in (**a**) and (**c**) represent 4 cm

To understand the roles of salt-inducible *OsbHLH035* during the seedling stage, we compared the plant growth and salt-stress responses in both WT and *Osbhlh035* seedlings under normal and salt-treated conditions. Under normal conditions, there were no significant differences in the growth patterns of WT and *Osbhlh035* aerial tissues on days 14 (Fig. 5a). Interestingly, there were also no obvious differences in the growth patterns and salt responses of the WT and *Osbhlh035* aerial tissues under salt-treated conditions (Fig. 5a). Moreover, the water-loss rate and ABA contents in both WT and *Osbhlh035* aerial tissues were comparable under either normal or salt-treated conditions (Fig. 5b and e). Furthermore, the primary root length and ABA contents of the *Osbhlh035* terrestrial tissues were similar to those of the WT terrestrial tissues under either normal or salt-treated conditions (Fig. 5c-e). These data indicate that OsbHLH035 does not appear to be involved in regulating plant growth during the seedling stage.

### Excess sodium accumulation in Osbhlh035 aerial tissues

**Fig. 6:**
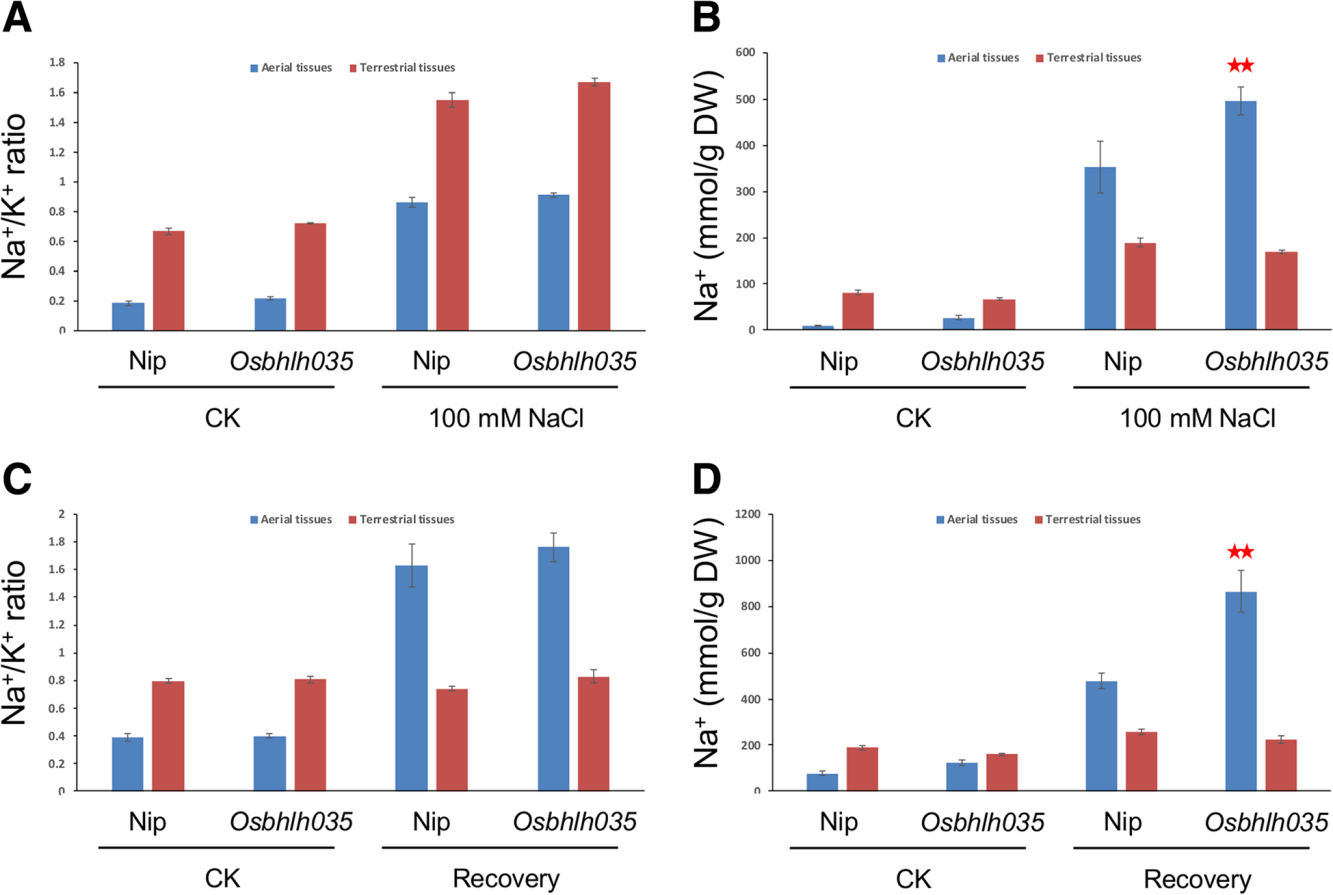
Effects of salinity on the Na^+^/K^+^ ratio and Na^+^ content in both WT and *Osbhlh035* seedlings. **a-b** Na^+^/K^+^ ratios and Na^+^ contents in aerial and terrestrial tissues from both salt-treated WT and *Osbhlh035* seedlings. **c-d** Na^+^/K^+^ ratios and Na^+^ contents in aerial and terrestrial tissues from both salt-relieved WT and *Osbhlh035* seedlings. The seedlings were grown on basal medium for 11 days and then transferred to basal medium containing 0 (CK) or 100 mM NaCl (salt-treated conditions) for an additional 3 days. After day 14, the seedlings were returned to basal medium for 7 days (Recovery). Asterisks indicate significant differences in comparison to WT (*P < 0.05 and **P < 0.01) based on Student’s *t*-test

**Fig. 7:**
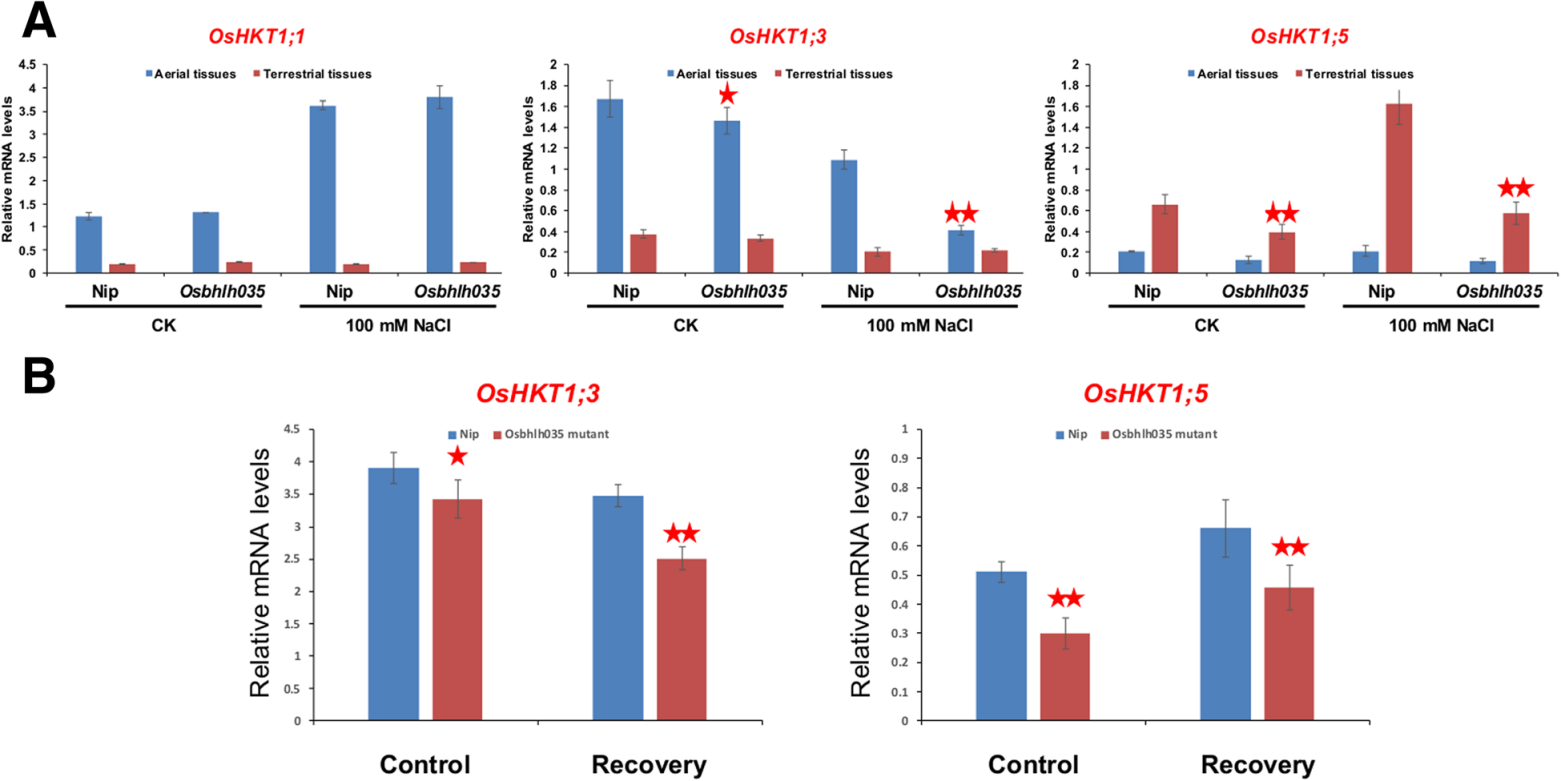
Effects of salinity on the *OsHKT1s* expression profile in both WT and *Osbhlh035* seedlings. **a** Quantification of *OsHKT1;1*, *OsHKT1;3*, and *OsHKT1;5* mRNAs in aerial and terrestrial tissues from both salt-treated WT and *Osbhlh035* seedlings. **b** Quantification of *OsHKT1;3* and *OsHKT1;5* mRNAs in aerial (left panel) and terrestrial (right panel) tissues, respectively, from both salt-relieved WT and *Osbhlh035* seedlings. The seedlings were grown on basal medium for 11 days and then transferred to basal medium containing 0 (CK) or 100 mM NaCl (salt-treated conditions) for an additional 3 days. After day 14, the seedlings were returned to basal medium for 7 days (Recovery). Asterisks indicate significant differences in comparison to WT (*P < 0.05 and **P < 0.01) based on Student’s *t*-test

As mentioned above, *OsbHLH035* is expressed in aerial and terrestrial tissues from the postgermination stage until the third-leaf stage (Fig. 2a), and its expression is enhanced by salt treatment (Fig. 1b). However, the roles of *OsbHLH035* do not appear to include regulating seedling growth or salt tolerance at these stages and under these conditions (Fig. 5). To investigate the roles of *OsbHLH035* in detail, we detected the sodium and potassium contents in both WT and *Osbhlh035* seedlings by using inductively coupled plasma-optical emission spectrometry (ICP-OES). As shown in Fig. 6a, the sodium/potassium (Na^+^/K^+^) ratios in the aerial or terrestrial tissues from the WT and *Osbhlh035* seedlings were comparable under either normal or salt-treated conditions; however, sodium was excessively accumulated in the *Osbhlh035* aerial tissues, while it was slightly reduced in its terrestrial tissues (Fig. 6b). These data suggest that Na^+^ exclusion from the transpiration stream is impaired in the *Osbhlh035* seedlings. Furthermore, q-PCR analysis revealed that *OsHKT1;3* and *OsHKT1;5* mRNA levels in *Osbhlh035* aerial and terrestrial tissues, respectively, were lower than those in the corresponding WT tissues under either normal or salt-treated conditions (Fig. 7a).

### OsbHLH035 contributes to seedling recovery following the removal of salt stress

**Fig. 8:**
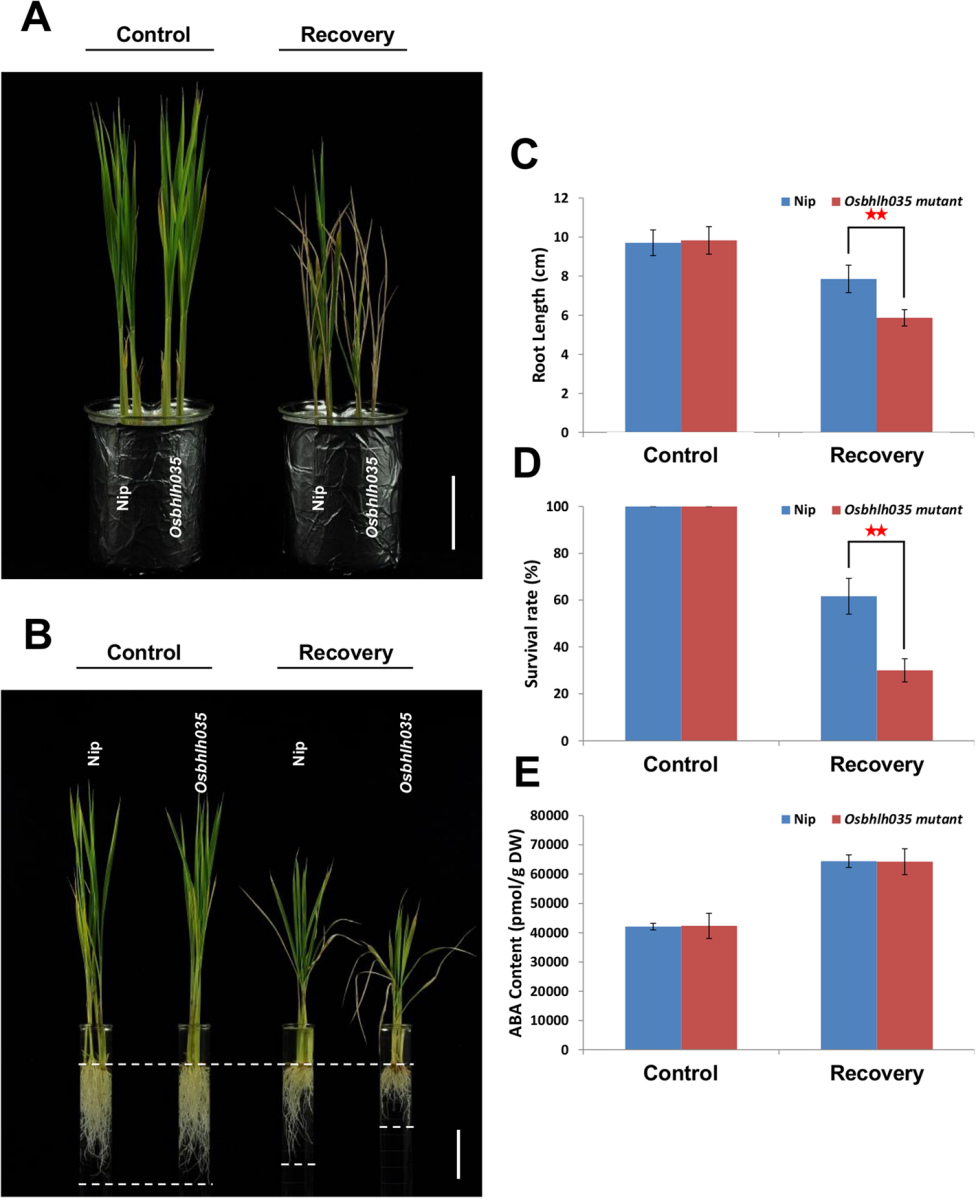
Investigating growth responses, survival rate, and endogenous ABA contents in both WT and *Osbhlh035* seedlings after recovery from salt stress. **a-b** The growth responses of aerial and terrestrial tissues in both WT and *Osbhlh035* seedlings after relief from salt stress. **c** The primary root lengths of WT and *Osbhlh035* seedlings after relief from salt stress. **d** The survival rate of WT and *Osbhlh035* seedlings after relief from salt stress. All survival rates are shown as the mean ± SD of two independent experiments (*n* = 20 × 3 independent biological replicates within each genotype and/or experimental condition). **e** The endogenous ABA contents of WT and *Osbhlh035* seedlings after recovery from salt stress. Scale bars in (**a**) and (**b**) represent 4 cm. The seedlings were grown on basal medium for 7 days and then transferred to basal medium containing 0 (control) or 100 mM NaCl (recovery) for an additional 7 days. After day 14, the seedlings were returned to basal medium for 7 days. Samples for the ABA ELISA were harvested on day 21. Asterisks indicate significant differences in comparison to WT (*P < 0.05 and **P < 0.01) based on Student’s *t*-test

As mentioned previously, the ability to tolerate and recover from stress are both important for rice to manage abiotic stresses (Lenka et al. 2011; Zhang et al. 2012). Therefore, we further investigated the growth responses of WT and *Osbhlh035* seedlings after the removal of salt stress. After a 7-day recovery period, new leaves sprouted in the WT seedlings but not in the *Osbhlh035* seedlings (Fig. 8a). The primary roots of WT seedlings that had recovered from salt stress were longer than those of the corresponding *Osbhlh035* seedlings (Fig. 8b, c). Moreover, the survival rate of WT seedlings after the removal of salt stress was approximately 61.67%, while the rate of *Osbhlh035* seedlings was only approximately 30% (Fig. 8d). Although the endogenous ABA content was still high in both the WT and *Osbhlh035* seedlings that had recovered from salt stress compared with controls, there were no significant difference between either the salt-stressed WT or *Osbhlh035* seedlings postrecovery (Fig. 8e). Notably, sodium was continuously over-accumulated in salt-relieved *Osbhlh035* aerial tissues (Fig. 6d). Moreover, the ratio of excessive Na^+^ in salt-relieved *Osbhlh035* aerial tissues is higher than that in salt-treated *Osbhlh035* aerial tissues (Fig. 6b vs. d). Besides, the expression of *OsHKT1;3* and *1;5* was also reduced in salt-relieved *Osbhlh035* aerial and terrestrial tissues, respectively (Fig. 7b). These data show that OsbHLH035 may play a positive, ABA-independent role in seedling recovery after salt stress.

### Delayed germination and impaired salt-stress recovery of Osbhlh035 mutants can be rescued via genetic complementation

**Fig. 9:**
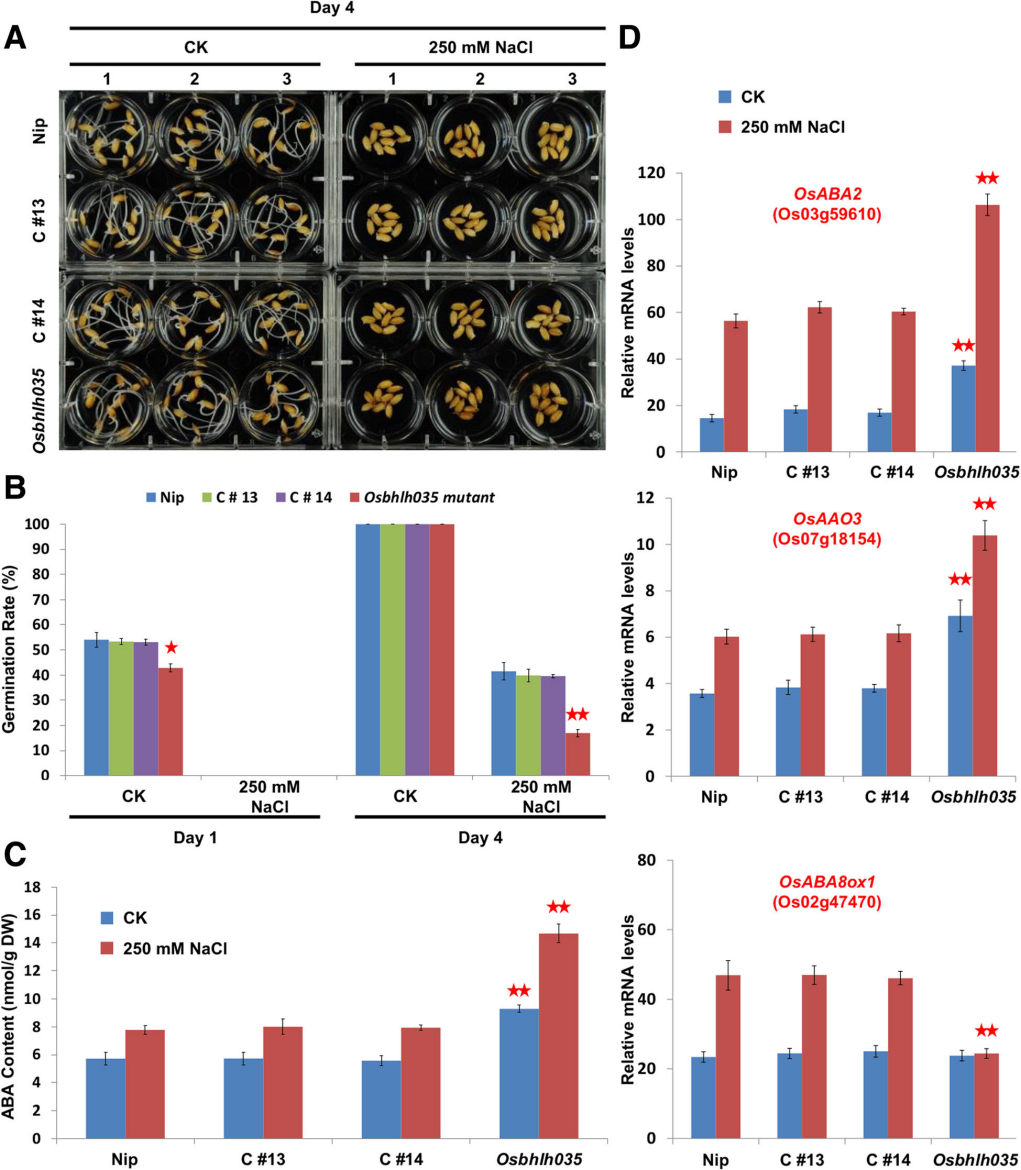
Examination of seed germination, endogenous ABA contents, and ABA metabolic gene expression in WT, *Osbhlh035*, and genetically complemented transformant rice. **a-b** The germination characteristics and rates of seeds from WT, *Osbhlh035*, and genetically complemented lines under normal and salt-treated conditions. Arabic numerals represent three independent biological replicates, which were harvested from three different rice plants per genotype. All germination rates are shown as the mean ± SD of two independent experiments; *n* = 50 × 3 biological replicates within each genotype and/or experimental condition. **c** The endogenous ABA contents of WT, *Osbhlh035*, and the genetically complemented transformant lines under normal and salt-treated conditions on day 4. **d** *OsABA2*, *OsAAO3*, and *OsABA8ox1* expression in WT, *Osbhlh035*, and the genetically complemented transformant lines under normal and salt-treated conditions on day 4. The seeds were grown on basal medium supplemented with 0 (CK) or 250 mM NaCl. Samples for the ABA ELISA and q-PCR assays were harvested on day 4. Asterisks indicate significant differences in comparison to WT (*P < 0.05 and **P < 0.01) based on Student’s *t*-test

Genetic complementation was performed to validate the roles of *OsbHLH035* in regulating seed germination and seedling recovery, and two representative complemented transformants (C#13 and C#14) were investigated. Compared with WT seeds germinated for 1 day under normal conditions or 4 days under salt-treated conditions, the corresponding *Osbhlh035* seeds displayed a delayed-germination phenotype with excess ABA accumulation on day 4 after the beginning of imbibition. However, the germination rate and ABA contents of the germinating C#13 and C#14 seeds were comparable to those of WT seeds (Fig. 9a-c). Moreover, *OsABA2*, *OsAAO3*, and *OsABA8ox1* expression in germinating C#13 and C#14 seeds was restored to levels similar to those of germinating WT seeds after 4 days under normal conditions or salt-treated conditions (Fig. 9d). These data demonstrate that OsbHLH035 plays a role in promoting germination by negatively regulating ABA-mediated seed germination.

**Fig. 10:**
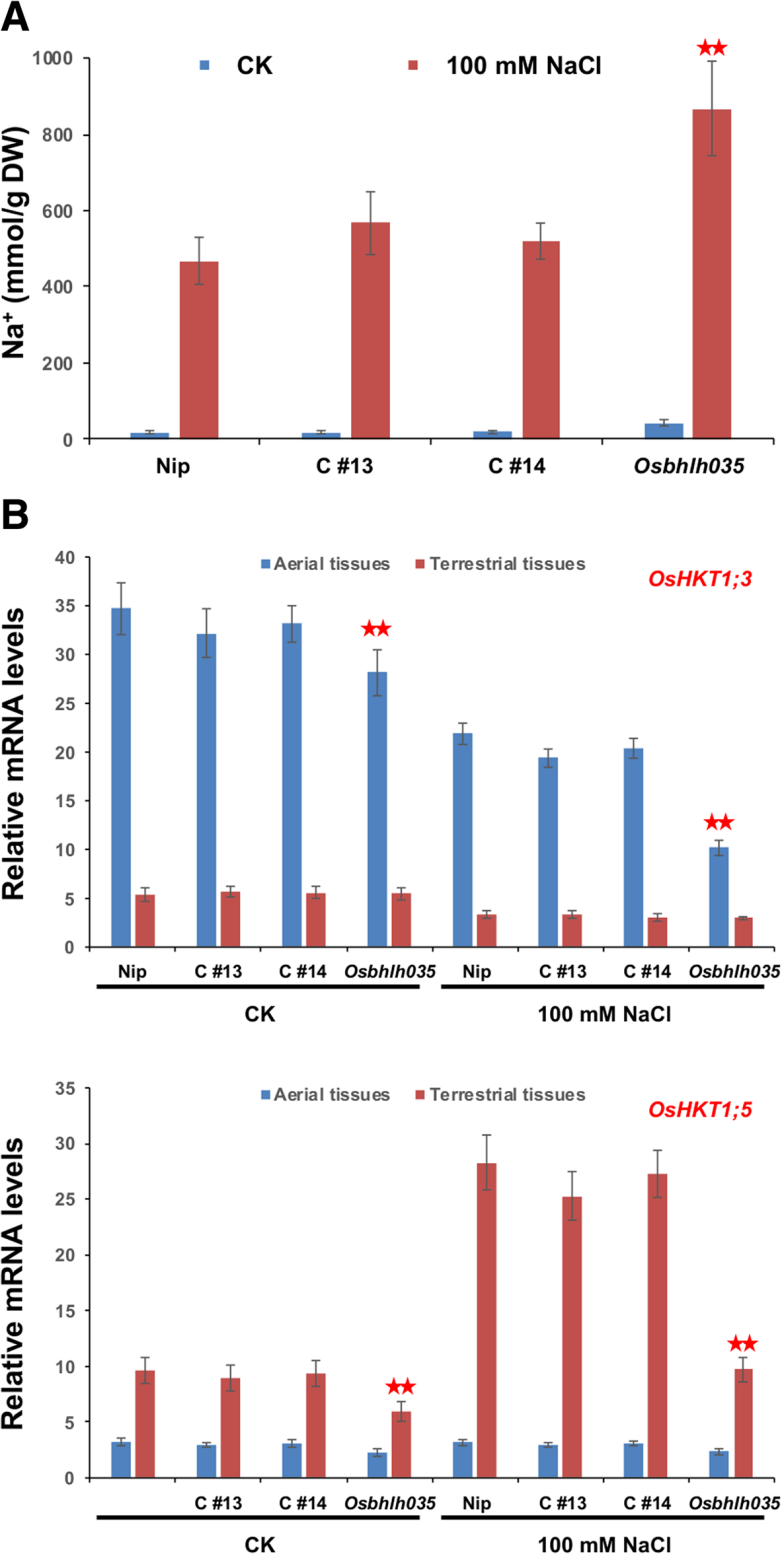
Na^+^ content **a** of the aerial tissues and the expression of *OsHKT1;3* **b** and *OsHKT1;5* **c** in WT, *Osbhlh035*, and genetically complemented transformant lines. The seedlings were grown on basal medium for 11 days and then transferred to basal medium supplemented with 0 (CK) or 100 mM NaCl for an additional 3 days. Asterisks indicate significant differences in comparison to WT (*P < 0.05 and **P < 0.01) based on Student’s *t*-test

**Fig. 11:**
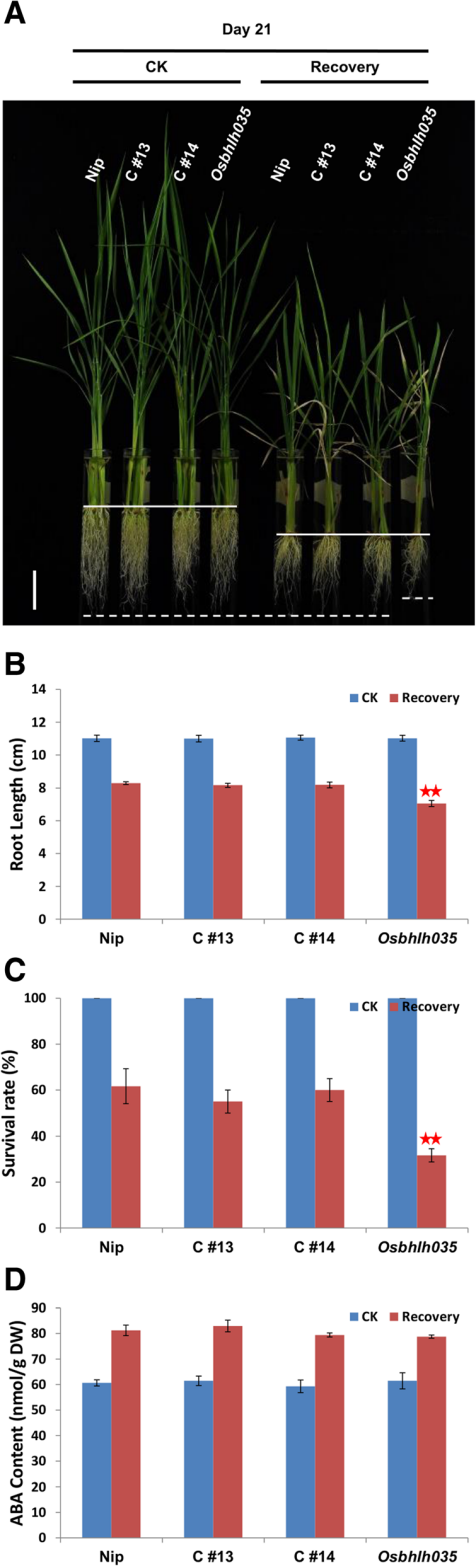
Phenotypic characterization and ABA contents after recovery from salt treatment. **a-d** Root elongation, survival rates, and endogenous ABA contents in WT, *Osbhlh035*, and genetically complemented transformant lines after salt stress removal. The seedlings were grown on basal medium for 7 days and then transferred to basal medium supplemented with 0 (CK) or 100 mM NaCl (recovery) for an additional 7 days, after which the seedlings were returned to basal medium for 7 days. Samples for the ABA ELISA and q-PCR assays were harvested on day 21. Asterisks indicate significant differences in comparison to WT (*P < 0.05 and **P < 0.01) based on Student’s *t*-test

Additionally, the excessive accumulation of sodium in *Obhlh035* aerial tissues and the impaired ability of *Osbhlh035* seedlings to recover from salt stress could also be rescued by genetic complementation. Under salt-treated conditions, the sodium levels in both the C#13 and C#14 aerial tissues were similar to those in corresponding WT tissues, with comparable levels of *OsHKT1;3* and *OsHKT1;5* expression (Fig. 10). After a 7-day recovery period, the primary root length and survival rates of both the C#13 and C#14 transformants were also comparable to those of WT seedlings (Fig. 11a-c). As expected, no obvious changes in endogenous ABA contents were observed between the WT, *Osbhlh035*, and two genetically complemented seedlings after 21 days under normal or post-salt-stressed conditions (Fig. 11d). These data suggest that OsbHLH035 might contribute to the ability of rice seedlings to recover from salt stress in an ABA-independent manner.

## Discussion

Plants are sessile and therefore must effectively cope with environmental changes to grow or survive. Thus, plants have evolved complicated gene regulatory networks, particularly diverse TFs, to respond to the surrounding environment. However, the functions of most TFs, as well as their involvement in plant growth, development and stress responses, are unknown. In this article, we conducted a functional study to address the roles of the TF *OsbHLH035* on seed germination, salt tolerance, and the ability to recover from salt stress in rice.

### OsbHLH035 mediates seed germination through an ABA-dependent pathway

ABA is important for coordinating the regulation of plant growth, development, and abiotic stress responses. However, the mechanisms that regulate the transcription of ABA metabolic genes in *Arabidopsis* and rice are poorly understood. In *Arabidopsis*, the ABA biosynthetic genes *AtNCED6* and *AtNCED9* contribute to ABA-mediated seed dormancy (Lefebvre et al. 2006). Notably, phylogenetic analysis reveals that plant NCEDs have undergone divergent evolution (Chen et al. 2011). In the rice genome, there are three putative NCEDs (see below for annotations) that are highly homologous to maize ZmVP14, the first plant NCED to be identified (Schwartz et al. 1997; Tan et al. 1997). To date, the rice orthologs of *AtNCED6* and *AtNCED9* have proven difficult to identify. In fact, both *OsNCED1* (Os03g44380) and *OsNCED3* (Os07g05940) expressions show no significant difference in germinating WT and *Osbhlh035* seeds, and *OsNCED2* (Os12g42280) transcripts are barely detectable under both normal and salt-treated conditions (data not shown). Apart from *AtNCEDs*, other biosynthetic genes such as *AtABA2* and *AtAAO3* as well as catabolic genes such as *CYP707A1/AtABA8ox1* and *CYP707A2/AtABAox2* are also involved in regulating seed germination. In germinating WT seeds, the expression levels of *OsABA2*, *OsAAO3*, and *OsABA8ox1*, which is a homolog of *CYP707A1*, *2*, and *3* (Additional file 1: Figure S5), are upregulated under salt-treated conditions (Fig. 4). However, under either normal or salt-treated conditions, both *OsABA2* and *OsAAO3* are more highly expressed in germinating *Osbhlh035* seeds than in corresponding WT seeds. No obvious differences in *OsABA8ox1* transcription were detected between germinating WT and *Osbhlh035* seeds under normal conditions, but the salt-induced expression of *OsABA8ox1* is abolished in germinating *Osbhlh035* seeds. Notably, the expression of *OsbHLH035-GFP* driven by a 2.0-kb *OsbHLH035* native promoter can restore *OsABA2*, *OsAAO3*, and *OsABA8ox1* expression and compensate for the germination deficiency in *Osbhlh035* mutants (Fig. 9). These data show that the excess ABA accumulation in *Osbhlh035* seeds germinating under salt stress is regulated at the transcriptional level due to not only the increased expression of ABA biosynthetic genes but also the inhibition of ABA catabolic gene expression. Given that OsbHLH035 is a G-box-binding TF, we searched for putative *cis*-acting elements in the upstream promoter regions of ABA metabolic genes. The 3807-bp and 3-kb regions upstream of the *OsAAO3* and *OsABA8ox1* initiation codons, respectively, were each found to contain one G-box element with a CACGTG core sequence. Because no G-box element was found in the promoter of *OsABA2* despite its increased transcription in the germinating *Osbhlh035* seeds, OsbHLH035 seems to suppress *OsABA2* expression in an indirect manner. Interestingly, a functional G-box element was identified to be necessary for drought-induced *AtNCED3* expression (Behnam et al. 2013). If this is the case, it cannot be excluded that OsbHLH035 suppresses *OsAAO3* and *OsABA8ox1* expression by interacting with G-box elements in their promoters. However, whether the transcriptional activity of *OsAAO3* and *OsABAox1* is directly regulated by OsbHLH035 needs to be confirmed.

### OsbHLH035 provides the ability to recover from salt stress in an ABA-independent pathway

In this article, our data showed that no obvious differences were observed in plant growth, water-loss rate, or endogenous ABA contents in both WT and *Osbhlh035* seedlings grown under normal or salt-treated conditions (Fig. 5). However, the *Osbhlh035* mutant accumulates an excess of Na^+^ ions in the aerial tissues and is unable to recover after salt stress relief (Figs. 6 and 8). These data indicate that OsbHLH035 seems to play a fine-tuning role in regulating salt stress response. Thus, the salt-susceptibility phenotype of *Osbhlh035* mutant becomes visible after salt stress relief. Besides, compared with that in aerial and terrestrial WT tissues, the expression of sodium transporter genes *OsHKT1;3* and *OsHKT1;5* is repressed in *Osbhlh035* tissues, respectively (Fig. 7). Notably, genetic complementation can restore *OsHKT1;3* and *OsHKT1;5* expression and rescue the impaired salt-stress recovery of the *Obhlh035* mutant (Figs. 10 and 11). These data suggest that OsbHLH035 confers the seedling recovery from salt stress through the ABA-independent activation of *OsHKT1*s. In *Arabidopsis* and rice, the HKT1s play a vital role in transferring Na^+^ from the xylem into xylem parenchyma cells (for review, see Su et al. 2015). This mechanism is used to prevent aerial tissues from Na^+^ over-accumulation and toxicity. Indeed, loss-of-function mutations in *OsHKT1*s, such as *OsHKT1;1* and *1;5*, lead to Na^+^ overaccumulation in the aerial tissues and display hypersensitivity to salt stress (Wang et al. 2015; Kobayashi et al. 2017). In fact, the NaCl-induced expression of *OsHKT1;1* is directly regulated by a TF, OsMYBc (Wang et al. 2015). *OsHKT1;3* and *OsHKT1;5* are mainly expressed in the aerial and terrestrial tissues of rice seedlings, respectively (Garciadeblas et al. 2003; Kobayashi et al. 2017). Although the expression of *OsHKT1;3* and *1;5* is downregulated in *Osbhlh035* aerial and terrestrial tissues, respectively, no G-box element was found in their 3-kb promoter. These data indicate that OsbHLH035 mediates the expression of *OsHKT1;3* and *1;5*, most likely in an indirect manner.

### Pleiotropic role of OsbHLH035 in regulating seed germination and salt-treated seedling recovery

In the plant *bHLH* TF gene family, the formation of homodimers or heterodimers is important for determining target gene expression. Dimeric molecules can specifically bind to certain *cis*-acting elements within the promoter regions of target genes, whose products can then mediate divergent physiological responses in plants. Therefore, plant bHLHs can play a dual or pleiotropic role in regulating growth and development based on their dimeric forms. For example, the POSITIVE REGULATOR OF GRAIN LENGTH 1-ANTAGONIST OF PGL1 (PGL1-APG) heterodimer, an OsHLH-OsbHLH complex, increases grain length and weight. However, the APG homodimer decreases grain length and weight (Heang and Sassa 2012). The function of OsbHLH068 not only is involved in regulating salt-stress responses but also may mediate flowering time in *Arabidopsis* (Chen et al. 2017). Thus, we assume that OsbHLH035 plays a pleiotropic role in regulating seed germination and seed recovery, presumably due to interactions with different TFs during the germination and seedling stages.

## Conclusions

In the present study, we propose that OsbHLH035 plays a pleiotropic role in regulating seed germination and salt-treated seedling recovery in rice. During the germination stage, OsbHLH035 alters the expression of ABA metabolic genes, which reduces ABA levels and relieves the inhibitory effect of ABA on germination. However, OsbHLH035 also mediates the expression of *OsHKT1* genes without altering ABA levels, which promotes seedling recovery from salt stress.

## Methods

### Plant materials, growth conditions, and experimental methods

Rice (*Oryza sativa* L.) cv. Nipponbare, an *Osbhlh035* mutant (*Tos17* line NG7221, obtained from the National Institute of Agrobiological Sciences [NIAS], Japan), and two independent transgenic complementation lines (C#13 and C#14, generated by *Agrobacterium*-mediated transformation) were analyzed in this study. Except in the germination test, the seeds used in all experiments were sterilized and imbibed at 37 °C for 2 days in the dark and then grown on a wire stand in a beaker at 28 °C under long-day conditions (16-h light/8-h dark cycle) with a light intensity of approximately 270 μE/s m^2^. For the germination test, seeds harvested from three independent plants were sterilized and grown directly in a Petri dish at 37 °C in the dark. The basal medium used in all of the experiments was Kimura B solution (Yoshida et al. 1976).

### RNA extraction, RT-PCR, and q-PCR

Total RNA was extracted from various rice tissues using an RNeasy Plant Mini Kit (Qiagen) according to the manufacturer’s instructions. To avoid genomic DNA contamination, up to 3 μg of total RNA was treated after extraction with Turbo DNA-*free*™ DNase (Ambion) following the manufacturer’s instructions, and 1 μg of DNA-free total RNA was then subjected to first-strand cDNA synthesis (Invitrogen, Cat No: 18080–051). The q-PCRs were carried out in an ABI 7500 system using the SYBR® Green PCR Master Mix Kit (Applied Biosystems [ABI]). The initial amount of template cDNA in each RT-PCR and q-PCR was 50 and 10 μg, respectively. At least three independent biological replicates were performed for each experiment, and *OsACTIN1* was used as an internal control for q-PCR normalization. The primer sequences used are provided in Additional file 2: Table S1.

### Water loss test

The detached aerial tissues from 14-day-old rice seedlings were placed in plastic weigh boats under ambient conditions. The fresh weight of the tissue was measured after 12.5 min and every 25 min thereafter for a total of 87.5 min. At least three independent biological replicates were performed for each experiment.

### ABA ELISA

Prior to the assay, the samples were vacuum dried at − 20 °C at least overnight for normalization to the final dry weight. ABA extraction, purification, and quantification were carried out as described previously (Lin et al. 2007), except that the samples were first ground using an SH-100 tissue homogenizer (Kurabo) before the addition of extraction buffer. At least three independent biological replicates containing two technical replicates each were performed for each experiment, and the data were presented as the average of two independent experiments.

### Measurement of ion contents

Samples were harvested, washed twice with ultrapure water, and subsequently vacuum dried at least overnight to measure the dry weight. The dried samples were digested using nitric and hydrochloric acids in an approximately 4:1 ratio and boiled at 200 °C for 2 h. Ion contents were determined using an inductively coupled plasma optical emission spectrometer.

### Transgene constructs and isolation of transgenic rice

The full-length coding sequences of *OsbHLH035* and *GFP* were PCR-amplified either with or without stop codons and cloned into the pGEM-T Easy vector. As shown in parenthesis below, these fragments were subcloned into the binary vector pCAMBIA-1300 where their expression was driven either by a 2.0-kb native promoter (*OsbHLH035::OsbHLH035-GFP* and *OsbHLH035::GFP-OsbHLH035*) or a CaMV 35S promoter (*35S::OsbHLH035-GFP* and *35S::GFP-OsbHLH035*). After the constructs were confirmed by sequencing, the *OsbHLH035::GFP-OsbHLH035* construct was transformed into the *Osbhlh035* mutant for subcellular OsbHLH035 localization and genetic complementation assays. Because the stable T_0_ rice transformants were chimeric and to avoid the undesirable side effects of multiple T-DNA insertions, T_1_ seeds were harvested from individual panicles of independent transformant lines that showed hygromycin resistance and sensitivity at a 3:1 ratio, normalized to seed viability, and used to further screen for homozygous transgenic rice. Additionally, the 2.0-kb *OsbHLH035* promoter was cloned into pCAMBIA-1305.1 (OsbHLH035*::*GUS) and transformed into the rice cv. Tainung 67 background for further investigation of the spatiotemporal expression of the *OsbHLH035* gene.

## Additional files

Additional file 1: **Figure S1.** Abiotic stress-responsive *OsbHLHs*. The fold change among each *OsbHLH* gene under different abiotic stresses is calculated as a ratio normalizing the data to it corresponding nontreated control. Red and green colors represent up- and downregulation, respectively. C, cold stress; D, drought stress; S, salt stress; and H, heat stress. **Figure S2.** Prediction of the conserved domains in OsbHLH035 and characterization of the NG7221 mutant line. (A) The ScanProsite tool in ExPASy (http://www.expasy.org/) predicts the presence of a typical bHLH domain (residues 64 to 113) in OsbHLH035. (B) Based on its annotation in the rice *Tos17* insertion mutant database (https://tos.nias.affrc.go.jp/), NG7221 is a single retrotransposon insertional line. (C) The identification of homozygous NG7221 mutants via PCR genotyping of gDNA. The primer positions and sequences are shown in Fig. 1a and Additional file 2: Table S1, respectively. Arabic numbers in (C) represent three independent biological replicates within each genotype. **Figure S3.** GFP-fused OsbHLH035 protein is predominantly localized to the nucleus in rice calli. The husk-removed seeds harboring *OsbHLH035::GFP-OsbHLH035* were placed on callus induction medium (Tran and Sanan-Mishra 2015) for 7 days and then the calli were subjected to GFP visualization by a confocal microscopy. Scale bars, 10 μm. **Figure S4.** The endogenous ABA contents in both germinating WT and *Osbhlh035* seeds. The husk-removed seeds were grown on basal medium for 2 days and then subjected to ABA ELISAs. **Figure S5.** Phylogenetic analysis of AtCYP707As and OsABA8oxs using the neighbor-joining method. Numbers next to the descendant indicate confidence values based on the bootstrap method. (PDF 570 kb)

Additional file 2: **Table S1.** Primers used in this study. (PDF 663 kb)

## Abbreviations

ABA: Abscisic acid
bHLH: Basic helix-loop-helix
GFP: Green fluorescent protein
GUS: β-glucuronidase
Nip: Nipponbare
q-PCR: Quantitative PCR
RT-PCR: Reverse transcription-PCR
TFs: Transcription factors
