# Supplementary material for: The transcription factor OsbHLH035 mediates seed germination and enables seedling recovery from salt stress through ABA-dependent and ABA-independent pathways, respectively
Source: Rice (N Y). 2018 Sep 10;11:50. doi: 10.1186/s12284-018-0244-z (PMC6134479; doi:10.1186/s12284-018-0244-z)
Supplement: Supplementary file 1 — Figure S1. Abiotic stress-responsive OsbHLHs. The fold change among each OsbHLH gene under different abiotic stresses is calculated as a ratio normalizing the data to it corresponding nontreated control. Red and green colors represent up- and downregulation, respectively. C, cold stress; D, drought stress; S, salt stress; and H, heat stress. Figure S2. Prediction of the conserved domains in OsbHLH035 and characterization of the NG7221 mutant line. (A) The ScanProsite tool in ExPASy (http://www.expasy.org/) predicts the presence of a typical bHLH domain (residues 64 to 113) in OsbHLH035. (B) Based on its annotation in the rice Tos17 insertion mutant database (https://tos.nias.affrc.go.jp/), NG7221 is a single retrotransposon insertional line. (C) The identification of homozygous NG7221 mutants via PCR genotyping of gDNA. The primer positions and sequences are shown in Fig. 1a and Additional file 2: Table S1, respectively. Arabic numbers in (C) represent three independent biological replicates within each genotype. Figure S3. GFP-fused OsbHLH035 protein is predominantly localized to the nucleus in rice calli. The husk-removed seeds harboring OsbHLH035::GFP-OsbHLH035 were placed on callus induction medium (Tran and Sanan-Mishra 2015) for 7 days and then the calli were subjected to GFP visualization by a confocal microscopy. Scale bars, 10 μm. Figure S4. The endogenous ABA contents in both germinating WT and Osbhlh035 seeds. The husk-removed seeds were grown on basal medium for 2 days and then subjected to ABA ELISAs. Figure S5. Phylogenetic analysis of AtCYP707As and OsABA8oxs using the neighbor-joining method. Numbers next to the descendant indicate confidence values based on the bootstrap method. (PDF 570 kb) [file 12284_2018_244_MOESM1_ESM.pdf]

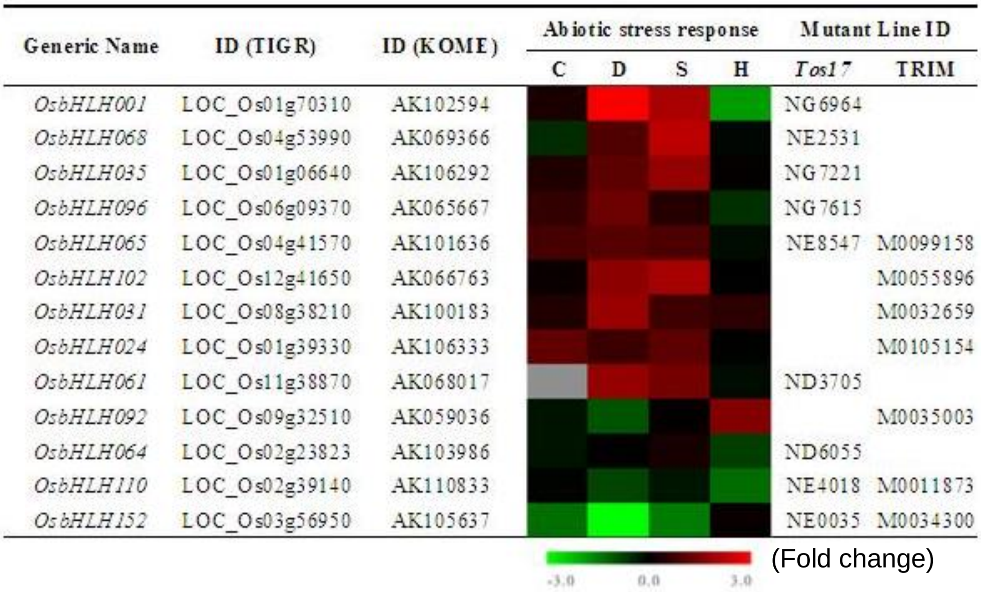

Generic Name	ID (TIGR)	ID (KOME)	Abiotic stress response				Mutant Line ID	
			C	D	S	H	*Tos17*	TRIM
OsbHLH001	LOC_Os01g70310	AK102594					NG6964	
OsbHLH068	LOC_Os04g53990	AK069366					NE2531	
OsbHLH035	LOC_Os01g06640	AK106292					NG7221	
OsbHLH096	LOC_Os06g09370	AK065667					NG7615	
OsbHLH065	LOC_Os04g41570	AK101636					NE8547	M0099158
OsbHLH102	LOC_Os12g41650	AK066763						M0055896
OsbHLH031	LOC_Os08g38210	AK100183						M0032659
OsbHLH024	LOC_Os01g39330	AK106333						M0105154
OsbHLH061	LOC_Os11g38870	AK068017					ND3705	
OsbHLH092	LOC_Os09g32510	AK059036						M0035003
OsbHLH064	LOC_Os02g23823	AK103986					ND6055	
OsbHLH110	LOC_Os02g39140	AK110833					NE4018	M0011873
OsbHLH152	LOC_Os03g56950	AK105637					NE0035	M0034300

Fig. S1 Abiotic stress-responsive *OsbHLHs*. The fold change among each *OsbHLH* gene under different abiotic stresses is calculated as a ratio normalizing the data to it corresponding nontreated control. Red and green colors represent up- and downregulation, respectively. C, cold stress; D, drought stress; S, salt stress; and H, heat stress.

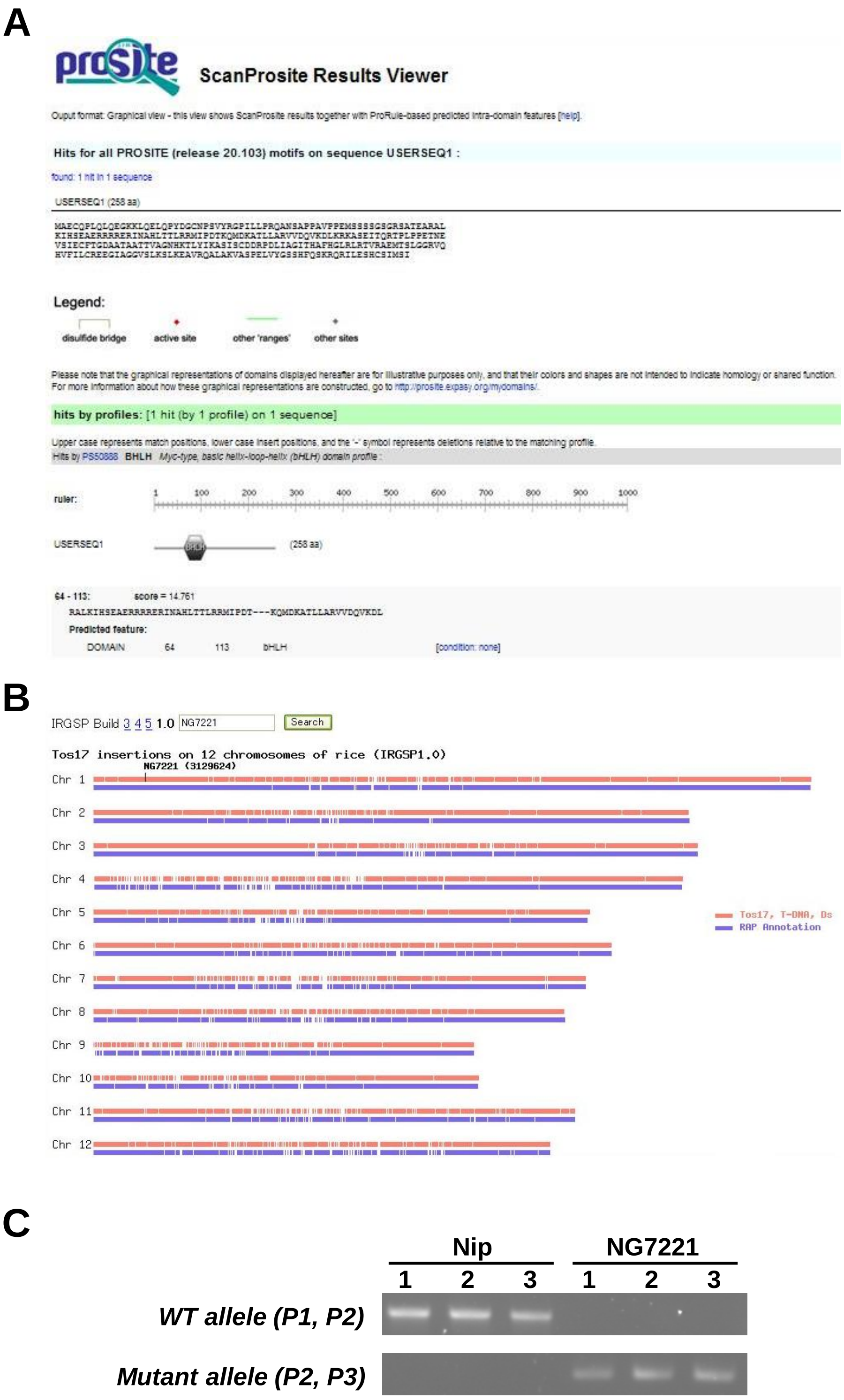

Fig. S2 Prediction of the conserved domains in OsbHLH035 and characterization of the NG7221 mutant line. (A) The ScanProsite tool in ExPASy (http://www.expasy.org/) predicts the presence of a typical bHLH domain (residues 64 to 113) in OsbHLH035. (B) Based on its annotation in the rice *Tos17* insertion mutant database (https://tos.nias.affrc.go.jp/), NG7221 is a single retrotransposon insertional line. (C) The identification of homozygous NG7221 mutants via PCR genotyping of gDNA. The primer positions and sequences are shown in Fig. 1A and Supplementary Table 1, respectively. Arabic numbers in (C) represent three independent biological replicates within each genotype.

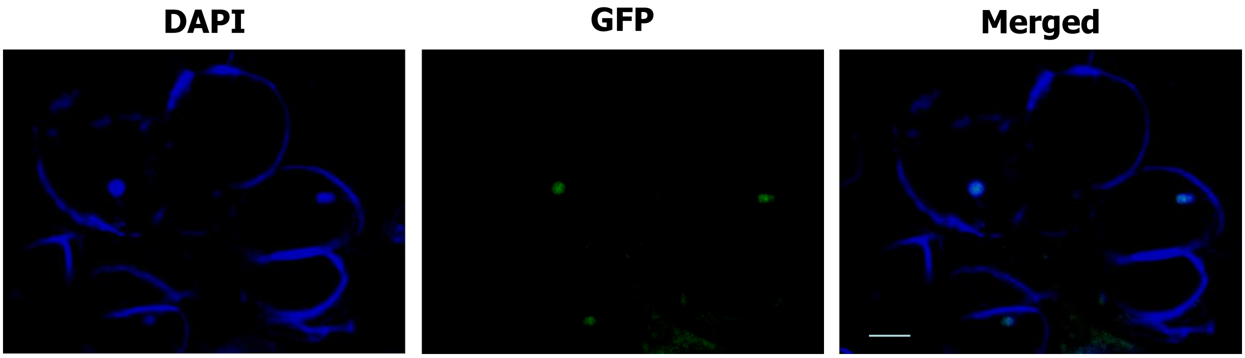

Fig. S3 GFP-fused OsbHLH035 protein is predominantly localized to the nucleus in rice calli. The husk-removed seeds harboring *OsbHLH035::GFP-OsbHLH035* were placed on callus induction medium (Tran and Sanan-Mishra 2015) for 7 days and then the calli were subjected to GFP visualization by a confocal microscope. Scale bars, 10 μm.

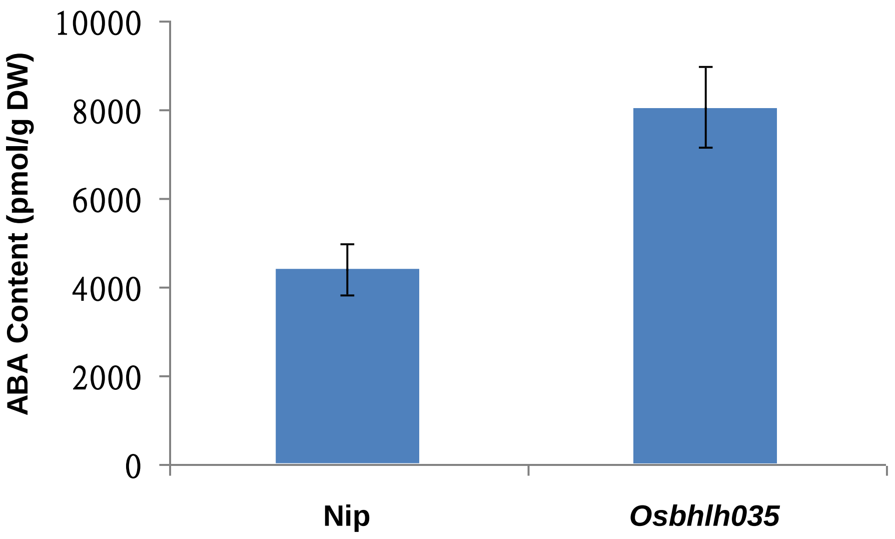

Fig. S4 The endogenous ABA contents in both germinating WT and *Osbhlh035* seeds. The husk-removed seeds were grown on basal medium for 2 days and then subjected to ABA ELISAs.

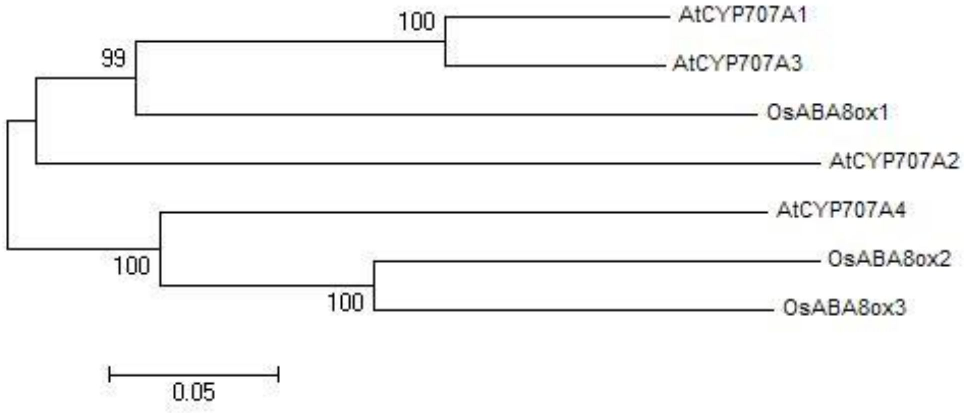

Fig. S5 Phylogenetic analysis of AtCYP707As and OsABA8oxs using the neighbor-joining method. Numbers next to the descendant indicate confidence values based on the bootstrap method.
