# Supplementary material for: The transcription factor OsbHLH035 mediates seed germination and enables seedling recovery from salt stress through ABA-dependent and ABA-independent pathways, respectively
Source: Rice (N Y). 2018 Sep 10;11:50. doi: 10.1186/s12284-018-0244-z (PMC6134479; doi:10.1186/s12284-018-0244-z)
Supplement: Supplementary file 2 — Table S1. Primers used in this study. (PDF 663 kb) [file 12284_2018_244_MOESM2_ESM.pdf]

Table S1 Primers used in this study.

PCR Type	Gene	Sequence (5'-3')	Length (bps)
gDNA genotyping	*OsbHLH035*	TTCGTTTTCCTTGCTTTGCT (P1)<br>CCTTCACTTGGTCCACCACT (P2)<br>ATTGTTAGGTTGCAAGTTAGTTAAGA (P3)	877 (P1, P2)<br>421 (P2, P3)
RT-PCR	*OsbHLH035*	CAGTGACAGCCGACAAAACCAGTTAGG<br>CAAGGTCTTGTGGTTGCCGGCGACAGTG	522
	OsACTIN1	GGAACTGGTATGGTCAAGGCTGG<br>CACACGGAGCTCGTTGTAGAAGG	252
q-PCR	*OsNCED1*	CTCACCATGAAGTCCATGAGGCTT<br>GTTCTCGTAGTCTTGGTCTTGGCT	221
	OsNCED2	GGTATGGAAACGAGGATAGTGGTT<br>TGCTTATTGTTGTGCGAGAAGTTC	197
	OsNCED3	CCCCTCCCAAACCATCCAAACCGA<br>TGTGAGCATATCCTGGCGTCGTGA	183
	OsABA2	GTGCAAGCAGCTCGAGCTTGAGC<br>AGCGCAACCTTGCTTTCCAACGG	173
	OsAAO3	GTGGAACAAGCTGTGCAAAGC<br>GCTAGTGCTGTCTGTGTTTCC	173
	OsABA8ox1	AAGCTGGCAAAACCAACATC<br>CCGTGCTAATACGGAATCCA	146
	OsHKT1;1	ATTAGCAGAGCACTGTGGAGGAA<br>CCGACGAACCCGTAGGAAG	245
	OsHKT1;3	CAGTTCATCTACCAAAACAATCCA<br>AATACCTCACCACCAATCAGCA	251
	OsHKT1;5	TGCCACCTTACACCACTTTCG<br>TGCCATACGCACTGATAACCTC	237
	OsACTIN1	CTCAGCACATTCCAGCAGATGTG<br>GATAACAGCTCCTCTTGGCTTAGC	126
